# Unexpected curious cause of serious air leakage after endotracheal intubation: A case report of tracheobronchomegaly and literature review

**DOI:** 10.3389/fsurg.2022.961186

**Published:** 2022-08-23

**Authors:** Jun Xiong, Quan Zhou, Yu Li, Yanyan Sun, Yajun Zhang

**Affiliations:** ^1^ Department of Anesthesiology, Shenzhen University General Hospital, Shenzhen University, Shenzhen, China; ^2^ Department of Radiology, Jiangsu Province Hospital of Integration of Chinese and Western Medicine, Affiliated Hospital of Integrated Traditional Chinese and Western Medicine, Nanjing University of Chinese Medicine, Jiangsu Province Academy of Traditional Chinese Medicine, Nanjing, China

**Keywords:** tracheobronchomegaly, Mounier-Kuhn syndrome, scoliosis, orthopedic, endotracheal intubation, difficult airway

## Abstract

**Purpose:**

Tracheobronchomegaly (TBM) is a rare disease with enlarged trachea and mainstem bronchi, which might not be diagnosed preoperatively because of patient’s nonsymptoms or clinicians’ overlook. These patients would be at fatal risk after general anesthesia endotracheal intubation due to severe peritubal leakage. This case may provide a helpful and informative resource for anesthesiologists and other clinicians, especially those managing patients’ airways.

**Clinical feature:**

We presented a patient undergoing elective scoliosis orthopedics who was postoperatively diagnosed with TBM. After general anesthesia endotracheal intubation, difficulty in maintaining ventilation with obvious peri-cuff air leakage made this rare disease to be suspected. The peritubal leakage was resolved by relocating the endotracheal tube to the subglottic area. Fortunately, there were no air leakage and postoperative complications.

**Conclusion:**

Anesthesiologists should keep the possibility of the unpredicted anatomic abnormal respiratory tract in mind, such as TBM.

## Introduction

Tracheobronchomegaly (TBM), also known as Mounier-Kuhn syndrome (MKS), is a rare clinical respiratory condition that was first reported by Mounier-Kuhn by detailed imageology and endoscopic examination in 1932 (1). The rare disease is commonly found in men with about 8:1 predominance with the characteristics of dilated trachea and/or proximal bronchi (2, 3). Patients may present with respiratory symptoms or recurrent respiratory tract infection; however, the clinical symptoms are not specific, from asymptomatic to severe respiratory failure (4). Although the etiology of TBM remains uncertain, the histological disorder is characterized by muscular and elastic tissue atrophy of the tracheobronchial tree, associated with eccentric diverticula and enlargement of the tracheobronchial tree (5). Because of its rare presentation and clinical polymorphism, there is a lack of enough attention to TBM. There were fewer than 400 cases reported outside China and just fewer than 30 cases reported in China (6, 7). We presented a scoliosis case with a postoperative diagnosis of TBM who encountered unexpected severe per-cuff leakage after tracheal intubation and mechanical ventilation. Anesthesiologists should be aware of an underlying TBM to avoid complications associated with the disease.

## Case presentation

A 42-year-old male patient with 161 cm height was admitted for ankylosing spondylitis and atlantoaxial dislocation. The patient presented pain and restricted movement in the lower back 15 years ago. He was definitely diagnosed with ankylosing spondylitis. Because of no specific and effective treatment, his sign of hunchback developed worse and worse. Three years ago, he presented neck stiffness with limited movement and pain in the occipital and nape region; meanwhile, his head leaned and fixed rightward. Because of the progressive condition, his trunk deformity developed into a more curve magnitude company with physical pain, which led to reduced quality of life and social and psychological distress. Therefore, he was admitted for spinal fusion and instrumentation surgery to restore the spine without neurological deficit.

The patient’s previous medical history was negative. He did not smoke and had no pets at home. There was no family history of any congenital, lung, or infectious conditions. After admission, he accepted a detailed assessment of neuromuscular spinal deformity, including clinical examination, cardiac and respiratory evaluation, and digestive and urinary assessment. Although there was thoracic deformity and motor disorder caused by ankylosing spondylitis, he did not present an obvious reduction in respiration and cardiac function. Also, there were no other apparent signs, for instance, abnormal chest auscultation. The preoperative thoracic computer tomography (CT) of the patient demonstrated multiple subpleural pulmonary bullae on the upper lobes of bilateral pulmonary.

Preoperative spirometry showed a forced expiratory volume in 1 s (FEV1) of 2.46 L (67.55% predicted), forced vital capacity (FVC) of 2.60 L (59.02% predicted), FEV1/FVC ratio of 113.07%, and a peak expiratory flow rate of 7.85 L/s (88.87% predicted).

**Figure 1:**
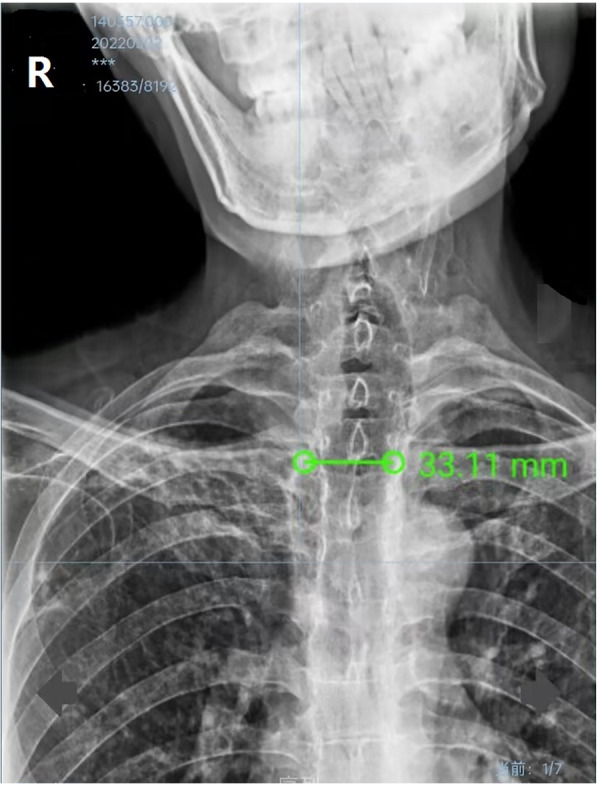
Preoperative thoracic digital radiography image showing that the transverse diameter of the trachea was 33 mm on the level of 2 cm above the aortic arch.

**Figure 2:**
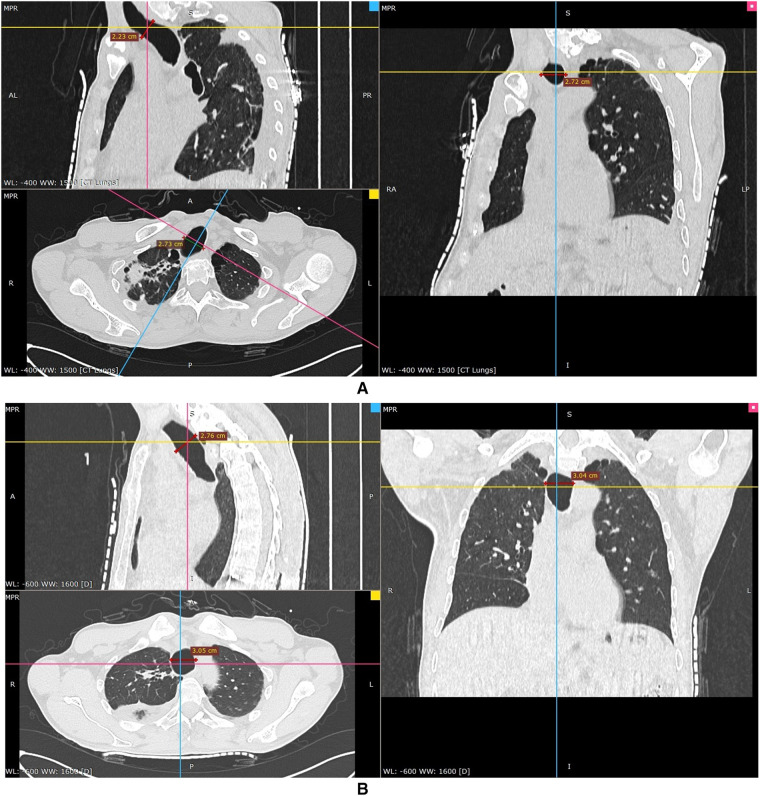
CT images of the lung demonstrating an enlarged trachea on the level of 2 cm above the aortic arch. The axial image (down-left) and coronal image (right) showed that the diameter of the trachea was 27.2 mm. The sagittal diameter (up-left) of the trachea was 22.3 mm (**A**). CT images of the lung on the level of the aortic arch, where the diameter of the trachea was the longest. The transverse diameter on the down-left axial image was 30.5 mm. The diameter on the right coronal image was 30.4 mm. Also, the sagittal image (up-left) demonstrated that the diameter of the trachea was 27.6 mm (**B**).

**Figure 3:**
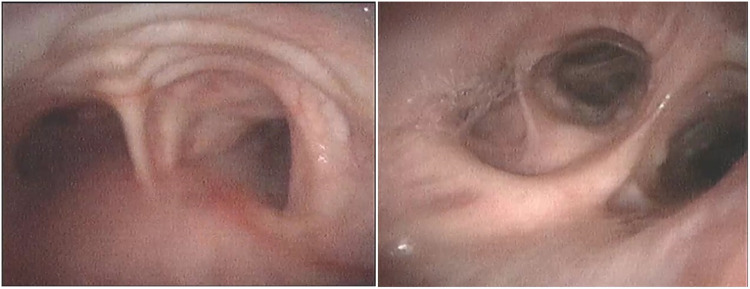
Bronchoscopy images showing that the mucosa protrudes between cartilaginous rings in trachea (left) and diverticula in the opening of the middle and lower lobes of the right lung (right).

After 1 month halo traction treatment, the patient was sent to the operating room for osteotomy orthopedics. Because his head was fixed rightward in the anterior flexion position, there was difficulty in rapid sequential induction; thus, awake nasotracheal intubation was implemented according to the preoperative anesthesia plan. After the standard monitoring was utilized and venous access was secured with an 18G cannula, the patient was administrated with 1 mg of midazolam and 5 µg of sufentanil, followed by ephedrine contracting vessel of the nasal mucosa and tetracaine nasopharynx mucosa surface anesthesia. During the process, the patient breathed with 5 L/min 100% oxygen under a mask covering his mouth and nose slightly. Meanwhile, he was administrated cricothyroid puncture with 5 ml of 1% tetracaine. After perfect surface anesthesia, a 6.0-size endotracheal tube (ETT) was inserted into the main trachea smoothly by a fiber bronchoscope guidance at 28 cm scale from the tip of the nose. Inflating cuff of ETT and connecting with breathing circuits. After the wave of end expiratory carbon dioxide was confirmed, the patient was administrated with 2 mg of midazolam, 20 µg of sufentanil, 100 mg of propofol, and 50 mg of rocuronium. When mechanism ventilation was used instead of spontaneous breathing completely, significant peritubular leakage happened accompanied by a sonorous sound from his mouth. At the same time, there was no distinct movement of the chest. An adequate seal was unable to achieve around the ETT cuff despite inflation with an increasing amount of air. Mechanism ventilation was quickly switched to manual ventilation to increase the volume of ventilation. There was still a sonorous sound of air leakage, but the movement of the chest and saturated pulse oxygen could be maintained. Bilateral respiration sounds were acquired by chest auscultation when manual ventilation. The ETT cuff was checked and injected with more air to stiff intensity. Fibrobronchoscope examination demonstrated the ETT located in the main airway again. Because of continuous air leakage and inability to maintain ventilation, the previous 6.0-size ETT was replaced by a 6.5-size ETT, and the cuff was inflated to stiff intensity. The situation made everyone disappointed due to uncorrected serious peri-cuff leakage. The patient’s preoperative chest digital radiography image and CT had to be reviewed further. His chest digital radiography image showed that a part of the main trachea was seemingly wider than normal (Figure 1). However, the widened main trachea was unable to be confirmed by chest CT because the images were not the standard due to his scoliosis profile. Meanwhile, fibrobronchoscopy examination had to be done again, and it demonstrated the tip of ETT close to the carina of the trachea; thus, the ETT was withdrawn 3 cm distance. The air leakage was corrected suddenly whenever manual or mechanical ventilation. Finally, the ETT was fixed at 25 cm from the tip of the nose, and the ETT cuff pressure was regulated suitably. There was no peri-cuff leakage until the end of the operation. After recovery from the operation, the patient accepted a thoracic CT scan again and a three-dimensional airway reconstruction. Meanwhile, the result of the postoperative X-ray tracheomalacia test was negative. TBM was considered by these standard radiological images (Figures 2, 3).

## Discussion

TBM is a rare disorder with the characteristic feature of an abnormally enlarged trachea and main bronchi, associated with tracheal diverticulosis, bronchiectasis, and recurrent respiratory tract infection. The etiology of this condition has not been understood clearly, but histological studies observe atrophy in smooth muscle and elastic tissue of the trachea and main bronchi (4). Except for congenital TBM, some other clinical conditions were also associated with secondary airway enlargement; thus, the change of this patient was suspected acquired because of no congenital respiratory disease but ankylosing spondylitis. Although clinical presentations of TBM are not specific, recurrent pulmonary infection is the most common symptom (6, 8, 9). This is the reason why most cases of TBM were reported with the presentation of pulmonary diseases. Nevertheless, there were much rarer TBM patients who underwent surgery and general anesthesia (GA) but without related clinical symptoms (10).

Although this rare condition is visible on plain chest X-ray, it is commonly overlooked on this conventional examination, even until a thoracic CT is done (11). This patient’s preoperative chest X-ray demonstrated an enlarged main trachea (Figure 1), but this was unfortunately overlooked. Because of his abnormal body profile, he thus accepted CT examination again after orthopedics to accurately evaluate the diameters of the trachea and main bronchus. The previous literature reported that the diameters of the normal trachea were 27 mm (sagittal section) and 25 mm (coronal section) in an adult male (12, 13). Thus, the radiological diagnosis criterion of TBM is defined by an increase in the transverse and sagittal diameters of the trachea beyond 25 and 27 mm, respectively (4). However, the tracheal diameter of Chinese adult males is much smaller than that of western adult males, whose transverse and sagittal diameters are only 21.48 and 21.55 mm, respectively (14). So, the patients’ diagnosis was definite. There are three subtypes of TBM based on the present anatomical classification of thoracic CT; this patient corresponded to the most common type II, which was tracheal dilatation abrupt transition to normal bronchi (15). Owing to this, it was impossible for the patient to maintain ventilation by simply inflating the ETT cuff, which was still unable to seal the enlarged trachea.

TBM might mainly produce difficulties for airway management because of the large and weak airways and other difficulties including ETT dislodgement, inefficient cough mechanism, and possible postoperative tracheal collapse (16). The significant air leakage of this patient after endotracheal intubation made us astonished. The maximal diameters of 6.0-size and 6.5-size ETT cuffs are 21 and 23 mm after inflation (Tuoren, Tuoren Medical Device Co., Ltd., Henan, China), respectively, which are significantly much shorter than the diameter of the patient’s dilated trachea. The curious trachea deformation was speculated when reviewing his plane chest X-ray. In fact, ETT cuff leakage during endotracheal intubation might be considered the first sign of TBM (17, 18).

The severe peri-cuff leakage not only causes trouble in maintaining the tide volume of mechanism ventilation but also increases the risk of pulmonary aspiration. The most prevalent method to reduce anesthesia gas leakage and respiration tract aspiration is to charge the throat with wet gauze (16, 19). Some TBM cases undergoing brain and cardiac operations were reported managing airway in this method (10, 20). However, it was unsuitable for this patient due to the prolonged operation and prone position. If cuff leakage took place during operation, it would be a disaster. A laryngeal mask is another recommendation to solve this airway management difficulty (19, 21). Nevertheless, the laryngeal mask is also inappropriate for this patient because of uncertain airway stability. Intraoperative dislocation of the laryngeal mask inevitably increases airway management difficulties in a prone position.

An additional recommended method is the subglottic placement of a tube cuff of large-size ETT to maintain tide volume and prevent pulmonary aspiration (20, 22, 23). In our case, by reviewing the thorax X-ray after anesthesia induction, the upper part of the trachea was likely normal; thus, the ETT was withdrawn till the cuff was located subglottic 1.5 cm. Cuff leakage was resolved perfectly with appropriate intracuff pressure. The postoperative thorax CT demonstrated that there was no suitable location for ETT cuff placement other than the subglottic area. Although large-size ETT or excessive inflation, even inflating 30 ml of air to 125 cmH_2_O intracuff pressure in 8.0-size ETT, may overcome the cuff leakage problem, it was possible to increase the risk of tracheal mucosal injury, even tracheal stenosis (10, 21). In patients with TBM, tracheal stenosis might take place following endotracheal intubation even with normal intracuff pressure (16). Therefore, some experts recommended cuffless ETT with oral gauze packing to create an airtight seal and avoid this possible complication (24).

Our case was fortunate because the surgery was completed smoothly without any postoperative complications. However, another case had to postpone surgery after endotracheal intubation due to this unexpected precarious, difficult airway, and epidural anesthesia was implemented instead of GA in the second same surgery (25). This may be a method when there is no method.

Thus far, there is limited consensus on GA airway management in patients with TBM. Region anesthesia is the first choice for these patients undergoing surgery of the abdomen and low limb (21). For patients with a preoperative diagnosis of TBM, an anesthesiologist should evaluate precisely the tracheobronchial diameter on a radiography scan and prepare a detailed management plan. In these patients, three-dimensional CT reconstruction of the trachea and bronchi is necessary to investigate the morphology of TBM and measure the trachea perpendicular to the longitudinal axis (10). In our case, the patient underwent the second surgery smoothly because of a precise evaluation and detailed anesthesia plan.

However, TBM could go undetected prior to GA because patients might be asymptomatic, or preoperative chest CT might not be routine. Even if chest radiological image is acquired, TBM could be easily overlooked, the same as in the present and previous cases (25). Generally, radiologists and anesthesiologists pay more attention to airway stenosis, but not airway dilatation or other obvious signs attract more attention from clinicians.

Inappropriate airway management in TBM patients during GA caused by preoperative overlook might bring these patients to fatal risk. Until now, there is a lack of airway management guidance for these TBM patients. If such patients need one-lung ventilation, their airway management would be a gigantic challenge for anesthesiologists due to no successful case of lung isolation by a double-lumen tube (21). Therefore, except for above-mentioned methods, a special designed ETT or tracheostomy tube would be needed if there was a tracheal anatomic abnormality, such as in patients with TBM, although it is seldom used. Sometimes anesthesiologists have to make airway tubes by themselves (19).

**Figure 4:**
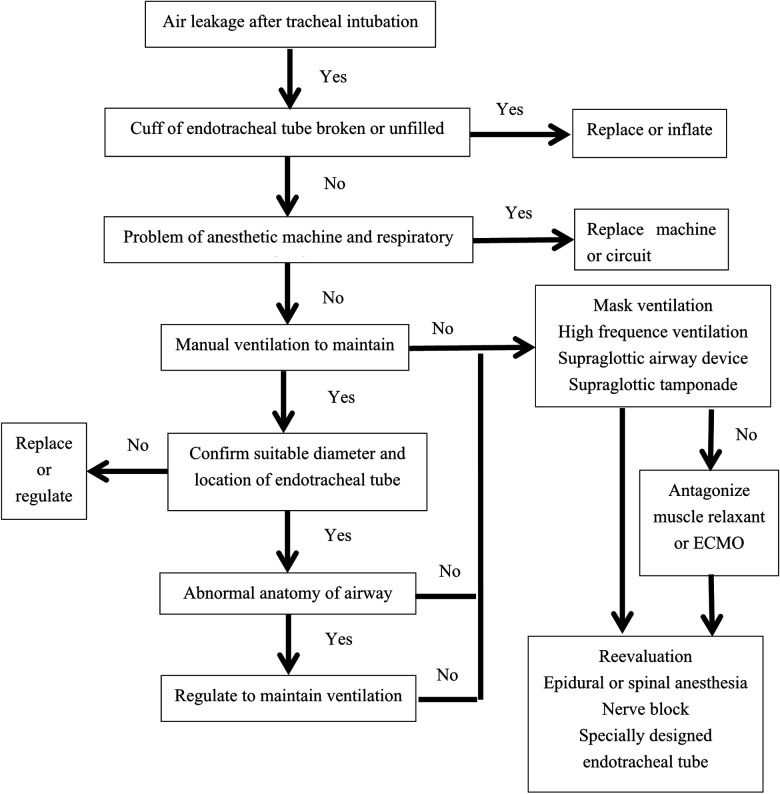
Protocol dealing with air leakage after endotracheal intubation. ECMO, extracorporeal membrane oxygenation.

Because respiratory diseases are not in our field, there were shortages of the introduction of TBM, such as its etiology, clinical and histological characteristics, and so on. Now, a protocol of dealing with air leakage after tracheal intubation was summarized (Figure 4); it might be helpful for anesthesiologists and other clinicians.

## Conclusion

Although TBM is a rare disease, patients with it might undergo GA or airway management in their life. Inappropriate airway management would produce related complications and even bring fatal risks. Preoperative diagnosis and precise evaluation are the best way to avoid these dangers. Clinicians should keep the possibility of TBM in mind, particularly when unexplainable peri-cuff leakage after endotracheal intubation. Until now, except for personal experiences, there is no guidance in airway management for these patients. Especially designed ETT according to these patients’ trachea anatomy might be a solution.

## Data Availability

The original contributions presented in the study are included in the article/**Supplementary Material**, further inquiries can be directed to the corresponding author/s.
